# Implicit and explicit learning in reactive and voluntary saccade adaptation

**DOI:** 10.1371/journal.pone.0203248

**Published:** 2019-01-16

**Authors:** Daniel Marten van Es, Tomas Knapen

**Affiliations:** 1 Behavioural and Movement Sciences, Vrije Universiteit Amsterdam, Amsterdam, Noord-Holland, the Netherlands; 2 Spinoza Centre for Neuroimaging, Royal Academy of Sciences, Amsterdam, Noord-Holland, the Netherlands; University of Muenster, GERMANY

## Abstract

Saccades can either be elicited automatically by salient peripheral stimuli or can additionally depend on explicit cognitive goals. Similarly, it is thought that motor adaptation is driven by the combination of a more automatic, implicit process and a more explicit, cognitive process. However, the degree to which such implicit and explicit learning contribute to the adaptation of more reactive and voluntary saccades remains elusive. To study this question, we employed a global saccadic adaptation paradigm with both increasing and decreasing saccade amplitudes. We assessed the resulting adaptation using a dual state model of motor adaptation. This model decomposes learning into a fast and slow process, which are thought to constitute explicit and implicit learning, respectively. Our results show that adaptation of reactive saccades is equally driven by fast and slow learning, while fast learning is nearly absent when adapting voluntary (i.e. scanning) saccades. This pattern of results was present both when saccade gain was increased or decreased. Our results suggest that the increased cognitive demands associated with voluntary compared to reactive saccade planning interfere specifically with explicit learning.

## Introduction

Successful interaction with the environment requires that motor actions precisely reach their intended targets. This poses a particularly challenging problem to the organism as conditions both within the body (e.g. waning or gaining of strength) and in the outside world (e.g. wind direction) are in constant flux. The problem is solved by ongoing calibration processes, referred to as motor adaptation. Studying the mechanisms involved in this continuous learning process is achieved by systematically influencing feedback to movements, such as saccades. Saccade adaptation can be induced by displacing the saccade target either closer to (leading to a decrease in saccade gain during adaptation) or further away from (leading to an increase in saccade gain) initial fixation during the executing of the saccade [1].

When saccades are evoked by sudden-onset peripheral stimuli, they are relatively fast and commonly referred to as reactive [2]. Alternatively, when saccades depend on behavioral goals they are relatively slow and often termed voluntary [3]. The adaptation of these types of saccades is thought to depend on different mechanisms [3]. The first evidence for this comes from findings of asymmetrical transfer of adaptation between saccade types [4–6]. Additionally, it was shown that voluntary and reactive saccades can be adapted simultaneously in different directions [7] and that adaptation of voluntary but not reactive saccades transferred to hand pointing motions [8].

The difference between reactive and scanning saccades can be well viewed as a differential cognitive involvement [9]. Although the exact functional link between attention and saccades remains elusive, different models of attention suggest a close relation [10–12]. Voluntary saccades especially demand the selective enhancement and suppression of visual information. In addition, voluntary saccades require the active maintenance of internalized stimulus-response rules in working memory. Indeed, voluntary saccade performance degrades with increasing working memory load [13], and depends on individual differences in working memory capacity [14].

Saccadic adaptation already occurs within the first few perturbation trials and continues throughout an experimental block. This learning also carries over to subsequent learning blocks, producing savings (i.e. quicker relearning), interference (i.e. slower learning of opposing adaptation) and spontaneous recovery (i.e. rebound of previous learning after forgetting [15,16]). These and other phenomena of motor learning can be well accounted for by a dual-state model of adaptation [17,18]. This model posits that learning is driven (1) by a fast process that learns and forgets quickly and (2) by a slow process that learns and forgets slowly. Recent studies have suggested that the fast process reflects the explicit and voluntary effort to actively counteract the perturbation (i.e. 'one-shot' learning), whereas the slow process reflects implicit and automatic learning [19,20]. In parallel to the distinction between reactive and voluntary saccades, it has been suggested that the difference between implicit and explicit learning can also be well understood in terms of differential cognitive involvement [21,22]. Specifically, it was shown that working memory capacity correlates with explicit and not implicit learning performance (in motor sequence learning [23], and in visuomotor adaptation [24]). In addition, it was shown that an interfering attentional task reduced the overall amount of learning during visuomotor adaptation [25], especially early during learning [26].

Together, the studies described above show that the difference between implicit and explicit learning on the one hand and between reactive and scanning saccades on the other hand can both be understood in terms of differential cognitive involvement. This raises the question to what extent implicit and explicit learning contribute to the adaptation of reactive and voluntary saccades. On the one hand, it is possible that the type of learning is contingent upon the mechanisms involved in saccade execution. This predicts that reactive saccade adaptation is driven predominantly by implicit, and voluntary saccade adaptation by explicit learning. On the other hand, and according to cognitive load theories of learning [27,28], the increased load associated with voluntary compared to reactive saccades could reduce remaining capacity required for explicit learning. This predicts the reverse relation where reactive saccade adaptation is driven predominantly by explicit, and voluntary saccade adaptation by implicit learning. In the present study, we aim to resolve these divergent predictions.

Another factor that potentially contributes to differential fast and slow learning is whether saccade amplitude is increased or decreased. After gain-down but not gain-up adaptation, saccade velocity and duration differ from unadapted saccades of equal amplitude. This has led to the suggestion that gain-down adaptation is driven by mid-flight corrections in the forward model, while gain-up adaptation is achieved by target remapping [29]. Such target remapping was shown to learn and forget slowly, while adaptions of the forward model learn and forget fast [30]. This implies that gain-down compared to gain-up adaptation should be driven more by the fast process.

Finally, awareness of the target displacement might additionally contribute to increased fast or slow learning and forgetting. From the viewpoint of error assignment [29], increased displacement awareness should cause errors for retinal error to be assigned to target motion. This in turn should lead to adaptation based on target remapping, and therefore to slow learning and forgetting [29]. From the viewpoint of explicit and implicit learning [19,20], increased displacement awareness should lead to increased explicit, and therefore fast learning and forgetting. These theoretical frameworks thus predict diverging relations between displacement awareness and fast and slow process contributions.

To test these hypotheses, we employed a global saccadic gain adaptation paradigm. This procedure induces strong adaptation that generalizes to all saccade directions [31]. During the execution of the saccade, the target was displaced either further away from (gain-up adaptation) or closer to (gain-down adaptation) previous fixation. Saccades were either elicited exogenously by a peripheral cue (reactive saccades), or endogenously by means of an internalized instruction to move to one of multiple targets (scanning saccades, a type of voluntary saccade). The path traveled across saccades was kept constant between the different saccade conditions. We assessed contributions of implicit and explicit learning as the gain of the slow and fast state respectively, as derived from the dual-state model of adaptation [17]. In addition, we assessed awareness of the target displacement on each trial using a ‘seen’ or ‘unseen’ judgement.

## Materials and methods

### Participants

Twelve participants (5 female) participated in this study. All participants gave written informed consent for participation. The study was approved by the ethical committee of the Vrije Universiteit Amsterdam.

### Apparatus

Stimuli were presented on a CRT monitor with a resolution of 1024x768 at a vertical refresh rate of 120Hz. Eye movements were recorded at 1000 Hz using an Eyelink 1000 Tower Mount (SR Research, Osgoode, Ontario, Canada). A 9-point calibration procedure was run before the start of each experimental condition. All light sources were eliminated in the experimentation room in order to minimize visual references. To eliminate remaining visible screen edges, we placed a red filter over the screen, only allowing red to pass through (+/- > 650 nm; Lee Filters color #787 'Marius Red'). All stimuli were consequently presented using only the red CRT channel. Thus, participants were in complete darkness and only viewed red stimuli.

### Task

**Fig 1:**
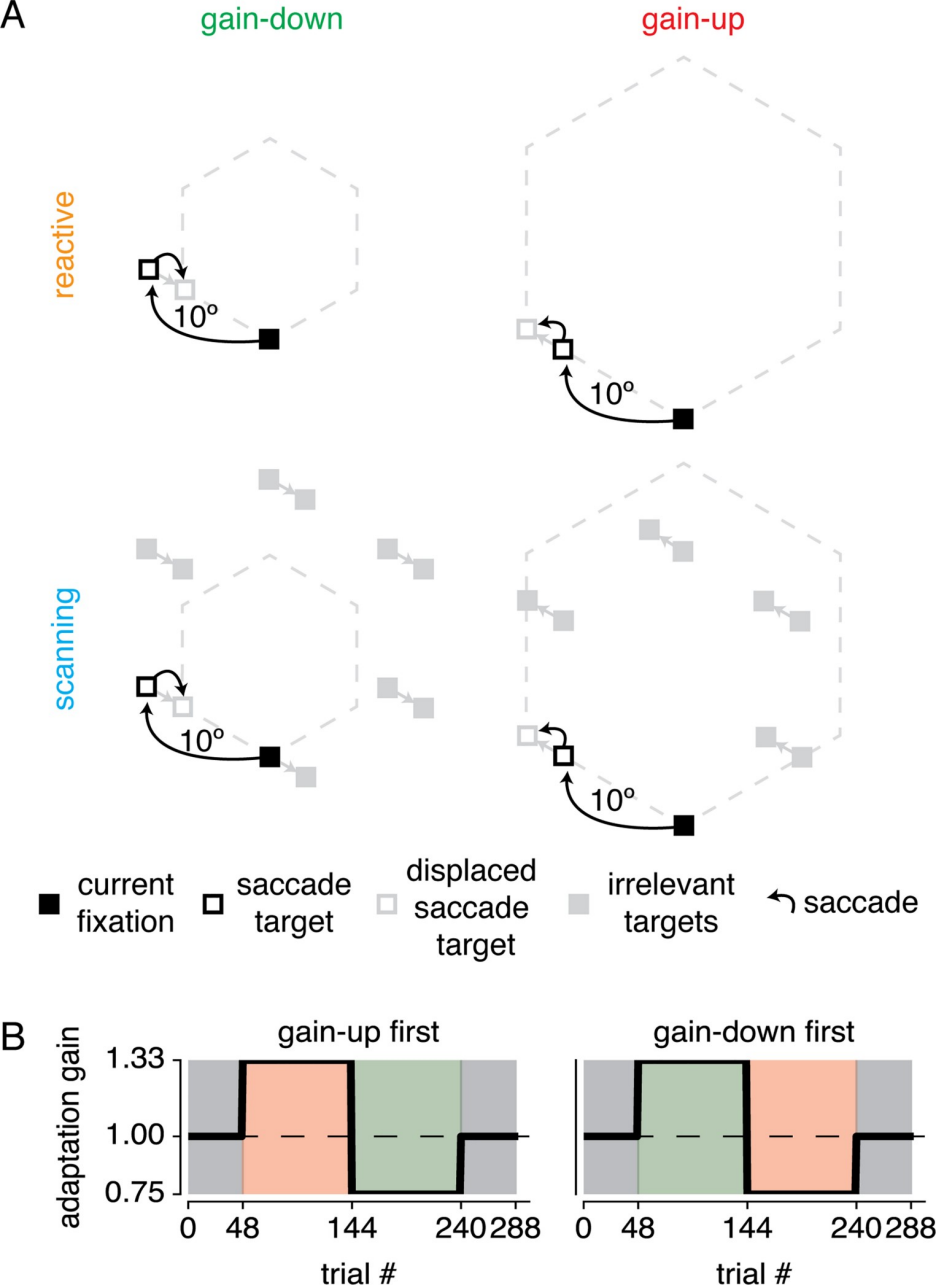
Experimental design. (A) Participants made saccades to targets around a clockwise hexagonal path. In the reactive saccade condition, a single target sequentially jumped along the corners of the hexagonal. Subjects were instructed to simply follow the target. In the scanning saccade condition, all targets were always presented and subjects were instructed to saccade to the next clockwise target when the currently fixated target flashed. During the saccade, targets jumped either further away from (gain-up) or closer to (gain-down) previous fixation. Participants sat in full darkness and all stimuli were filled red squares. Note that dotted outline and different appearance of squares are for illustrative purposes only. (B) The experiment was made up of an initial baseline block, two adaptation blocks and a final baseline block. All participants completed two experimental sessions per saccade type, switching the adaptation block orders between gain-up and gain-down.

Participants followed a square target (0.5x0.5 dva) around the screen along a clockwise hexagonal path, as depicted in Fig 1A. Participants started each block fixating at the central and lower position. On all trials in all conditions, the currently fixated target flashed for 150 ms to indicate the eye movement instruction. In the reactive saccade condition the current fixation target extinguished and immediately appeared in the next location along the hexagonal, provoking a peripheral visual transient. In the scanning saccade condition all possible locations along the hexagonal were always present on the screen, meaning there were no peripheral visual transients before the saccade. Importantly, in both saccade conditions participants completed identical saccade paths. The only difference was the number of presented targets (i.e. only one or all). This ensured that in the reactive saccade conditions subjects could simply follow the target around the screen, whereas in the scanning saccade conditions participants had to actively select one of multiple targets and plan a saccade to it. As soon as the initial saccade was detected by experimental software, the target(s) displaced either 3.3 dva further along the saccade trajectory (gain-up adaptation), or 2.5 dva back in the opposite direction of the saccade trajectory (gain-down adaptation). These differential absolute displacement magnitudes ensured equal relative gains (7.5/10 ≈ 10/13.3). Thus, in the reactive saccade condition only the single target was displaced, whereas in the scanning condition all targets displaced simultaneously.

To ensure unpredictability of the saccade target onset time, the fixation interval was randomly selected from an exponential distribution with minimum of 250 ms and mean of 550 ms. Subsequently, the currently fixated target flashed (150 ms) after which polling for saccades started (using threshold of 1.5 dva; maximally 2750 ms). As soon as the saccade was detected, the saccade target was displaced.

To asses awareness of the target displacement, participants indicated on each trial whether they perceived the target displacement. For this, the square saccade target turned into a triangle that pointed either left- or rightwards, 500 ms after saccade target displacement. Participants were instructed to press in the direction of the triangle to report the displacement as 'seen' and in the opposite direction to indicate 'unseen'. The response window ended as soon as a response was given or when 2000 ms passed. No feedback was provided.

### Procedure

Each experimental condition was made up of four blocks (see Fig 1B). In the first and last baseline blocks of 48 trials no target displacements took place to establish baseline measurements of saccade parameters. The second and third blocks contained 96 trials of either gain-up or gain-down adaptation. During the course of an adaptation block, participants learned the regularity in the target displacement and gradually adapted the gain of their initial saccade to land closer to the post-displacement location. In total, there were four experimental sessions (i.e. full factorial design with factors saccade type [reactive/scanning] and adaptation direction order [up-down/down-up]). Each experimental session lasted approximately 10 minutes. Before the first experimental session, participants practiced a shortened version of the experiment (6 saccades per block). This allowed them to estimate expected jump magnitude to judge as 'seen' or 'unseen' and to practice with the task procedure in general. We took several precautions to ensure that lingering adaptation between different sessions [32] would not influence our results. First, the different experimental sessions were separated by at least 2 hours. Second, the order of the four conditions were randomized on the factor of saccade type, yielding 12 possible orders (4!/2) each assigned to a different participant. Third, the first block recorded baseline saccade gain. We report adaptation gain relative to this baseline measure.

### Data analysis

#### Saccade amplitude definition

Eyetracking data were preprocessed using the hedfpy package (https://github.com/tknapen/hedfpy). Offline saccade detection was performed using the Engbert and Mergenthaler algorithm [33]. The preprocessed data and the analyses presented in this manuscript can be found under https://figshare.com/s/2d97ad68b6ec3801314c and https://github.com/daanvanes/implicit_explicit_SA respectively. Saccade amplitudes were converted to 'gain' by dividing them by the median saccade amplitude from the first baseline block, for each of the 6 hexagonal directions separately. Trials were rejected when (1) it included a blink, (2) saccade amplitude was below 3 dva or more than 3 two-sided median absolute deviations in that block and (3) saccade start point deviated more than 1.5 dva from the pre-saccadic target.

#### Exponential fits to second block

In order to quantify the timescale of adaptation in the first adaptation block we fitted exponentials of the form:

$$fit=(1-\alpha )∙(e^{-t\beta }-1)+1$$ (1)

where *α* and *β* reflect a gain and timescale parameter respectively and *t* reflects trial number. This form of the exponential equation ensures that it starts at 1 and approaches (1 − *α*) at infinite *t*. Including the gain parameter (*α*) ensures that the estimate of the timescale (*β*) is unaffected by overall adaptation magnitude. Estimates for the reliability of parameters in this function were found by using a 10^5^ fold bootstrap procedure. Within each fold of this procedure, the exponential function was fitted using least-squares to average data of a particular condition across a random sample of participants with replacement (cf. Fig 4A and 4B). In order to retrieve differences between conditions, exponential functions were fitted to all relevant conditions within each fold, ensuring paired comparisons between bootstrap samples of participants (cf. Fig 4C). Resulting p-values were calculated as the ratio of parameter difference estimates that fell below versus above 0, multiplied by 2 (i.e. two-tailed tests).

#### Fast and slow process contribution

In order to determine the fast and slow process contributions to reactive and scanning saccade adaptation, we fitted the multi-rate model [17] to the data. We set learning and retention parameters to the values established in the original study (fast learn = .21, fast retention = .59, slow learn = .02 and slow retention = .992), and varied gain parameters that scaled the contribution of each process. We fixed the learning and retention parameters in order to maximize stability of our parameters of interest (i.e. the gain parameters). Thus, adaptation was given by the following:

$${state}_{fast}^{\left(i\right)}=\alpha_{fast}∙\varepsilon^{\left(i\right)}+\beta_{fast}∙{state}_{fast}^{\left(i-1\right)}$$ (2)

$${state}_{slow}^{\left(i\right)}=\alpha_{slow}∙\varepsilon^{\left(i\right)}+\beta_{slow}∙{state}_{slow}^{\left(i-1\right)}$$ (3)

$$adaptation=\gamma_{fast}∙{state}_{fast}+\gamma_{slow}∙{state}_{slow}$$ (4)

where *α*, *β*, *γ*, *ϵ* and *i* refer to learning rate, retention rate, process gain, saccade error and trial index respectively.

We also fitted the model using freely varying learning and forgetting parameters. This yielded similar results, although parameter estimates were less stable. This is likely caused by the fact that parameter estimates trade off in the fitting procedure. This interaction between model parameters is illustrated by the following. When learning is relatively strong and forgetting relatively weak, this results in greater overall learning. Similarly, increasing the gain parameter while keeping the learning and forgetting rates equal also results in greater overall learning. Although this latter approach fixes the shape of the individual slow and fast processes, it does allow for the shape of overall learning to vary as a result of differential weighing of the fast and slow processes. As we are specifically interested in the shape of the overall learning curve and not in the shape of either the fast or slow processes alone, we opted to fix the learning and forgetting parameters in order to maximize the stability of the gain parameters. Future studies interested in differences in the shape of fast and slow learning could include error-clamp trials, where visual error is eliminated by displacing the saccade target to the saccade endpoint [18].

## Results

**Fig 2:**
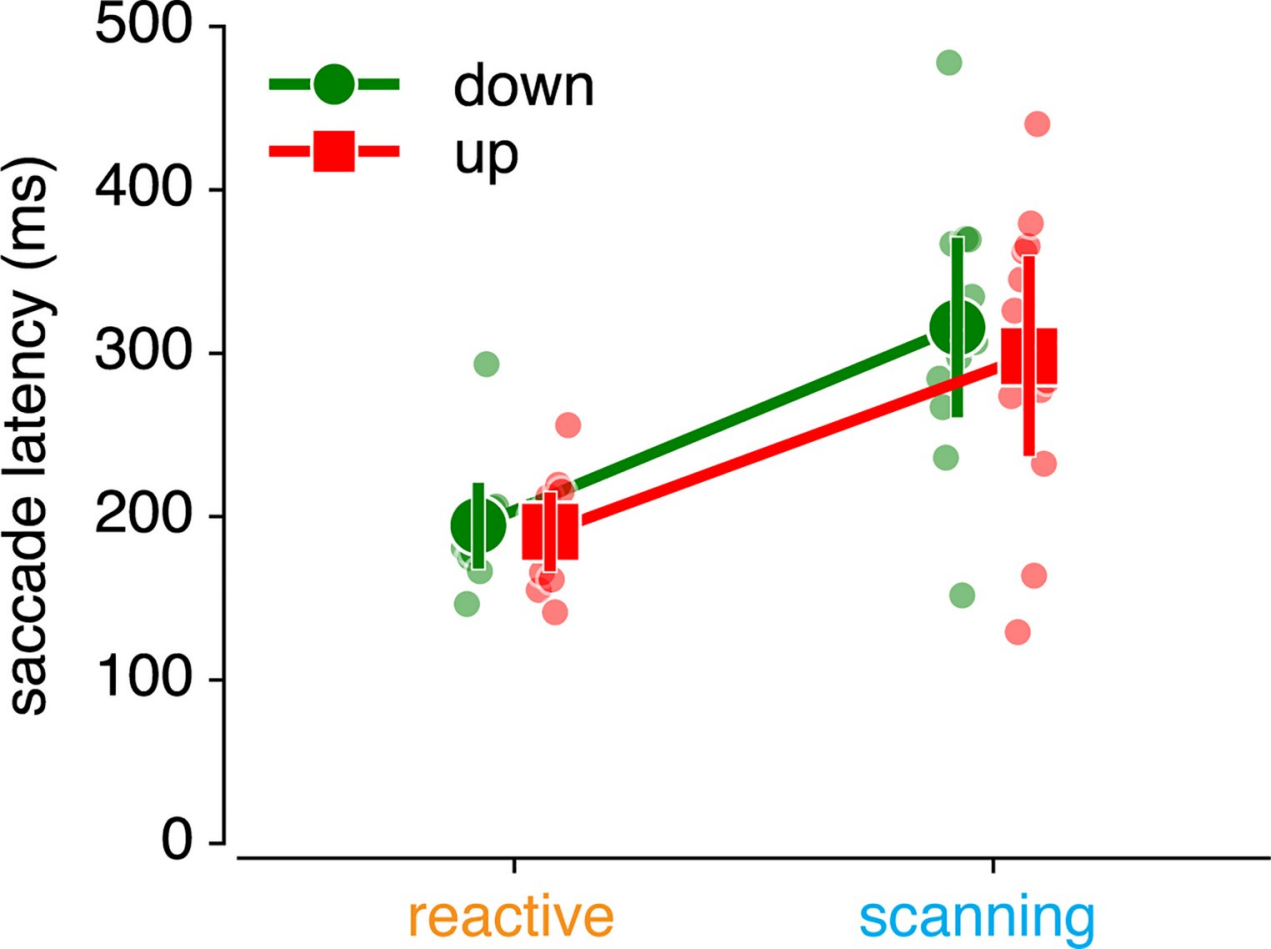
Saccade latencies. Saccade latencies in the second block of each saccade adaptation condition show that scanning saccades are slower than reactive saccades.

First, we verified whether we successfully evoked reactive and voluntary saccades by investigating their relative latencies. Reactive saccade latency is usually around 200 ms, whereas voluntary saccade latency commonly exceeds 250 ms [3]. Our results indeed showed that reactive compared to scanning saccades were faster (193 vs. 307 ms; see Fig 2; F_(1,11)_ = 34.348, p = 1.092 * 10^−4^, *η*^2^*p* = .833). In addition, saccade latency was not different for gain-up vs. gain down adaptation (F_(1,11)_ = 1.010, p = .337, *η*^2^*p* = .007), nor was there an interaction between saccade type and adaptation direction (F_(1,11)_ = 0.292, p = .600, *η*^2^*p* = .003)).

**Fig 3:**
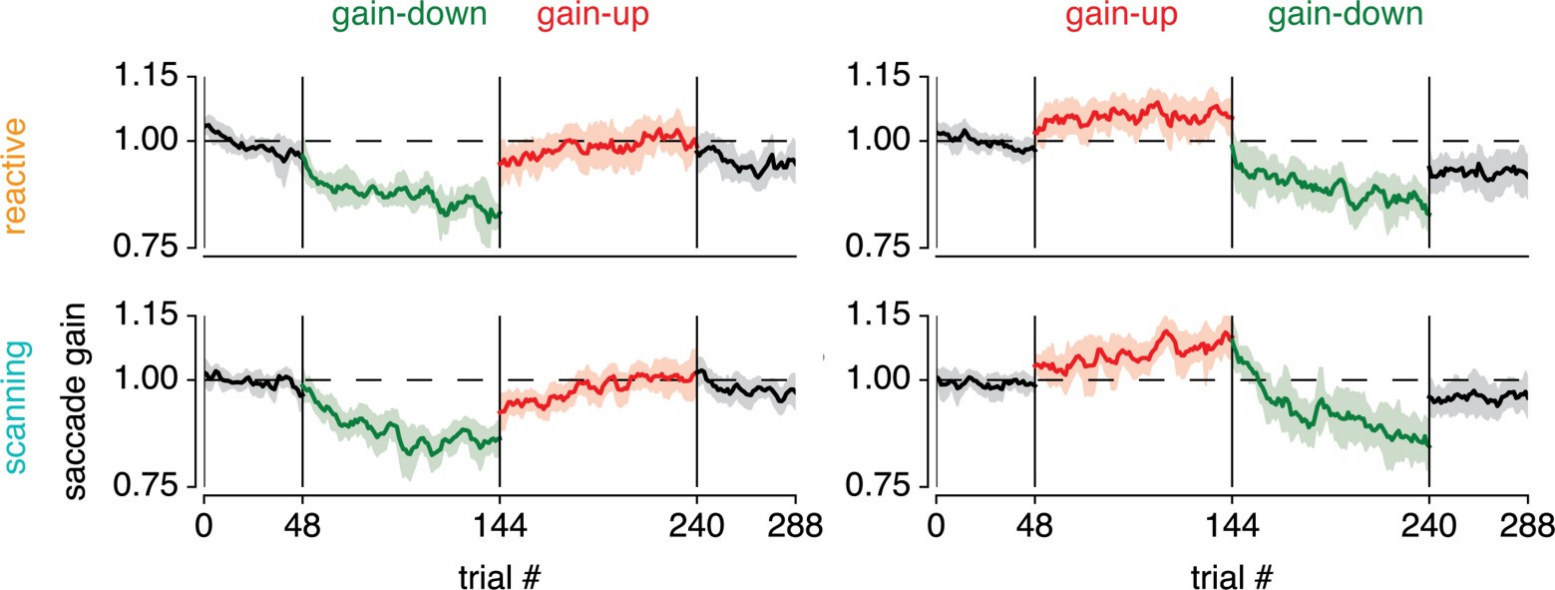
Saccade adaptation time-courses for the different conditions. Saccade amplitude across trials for the different saccade conditions (reactive and scanning) and for the different orders of gain direction blocks (down-up or up-down). Saccade amplitude is depicted as the ratio to median saccade amplitude in the first block (per saccade direction, see Methods) and shown as running average over 6 saccades (the hexagonal period). Shaded areas correspond to 95% CI over participants.

Fig 3 shows saccade amplitude across trials and highlights several features. First, and in correspondence with the literature, it shows stronger gain-down compared to gain-up adaptation [34–37]. In addition, it shows that adaptation of scanning and reactive saccades is of comparable magnitude. Second, it suggests that reactive saccade adaptation reaches maximal adaptation early in the block, whereas scanning saccade adaptation continues more strongly throughout the block. Finally, adaptation seems to change more strongly between blocks 2 and 3 in the reactive compared to the scanning saccade conditions.

**Fig 4:**
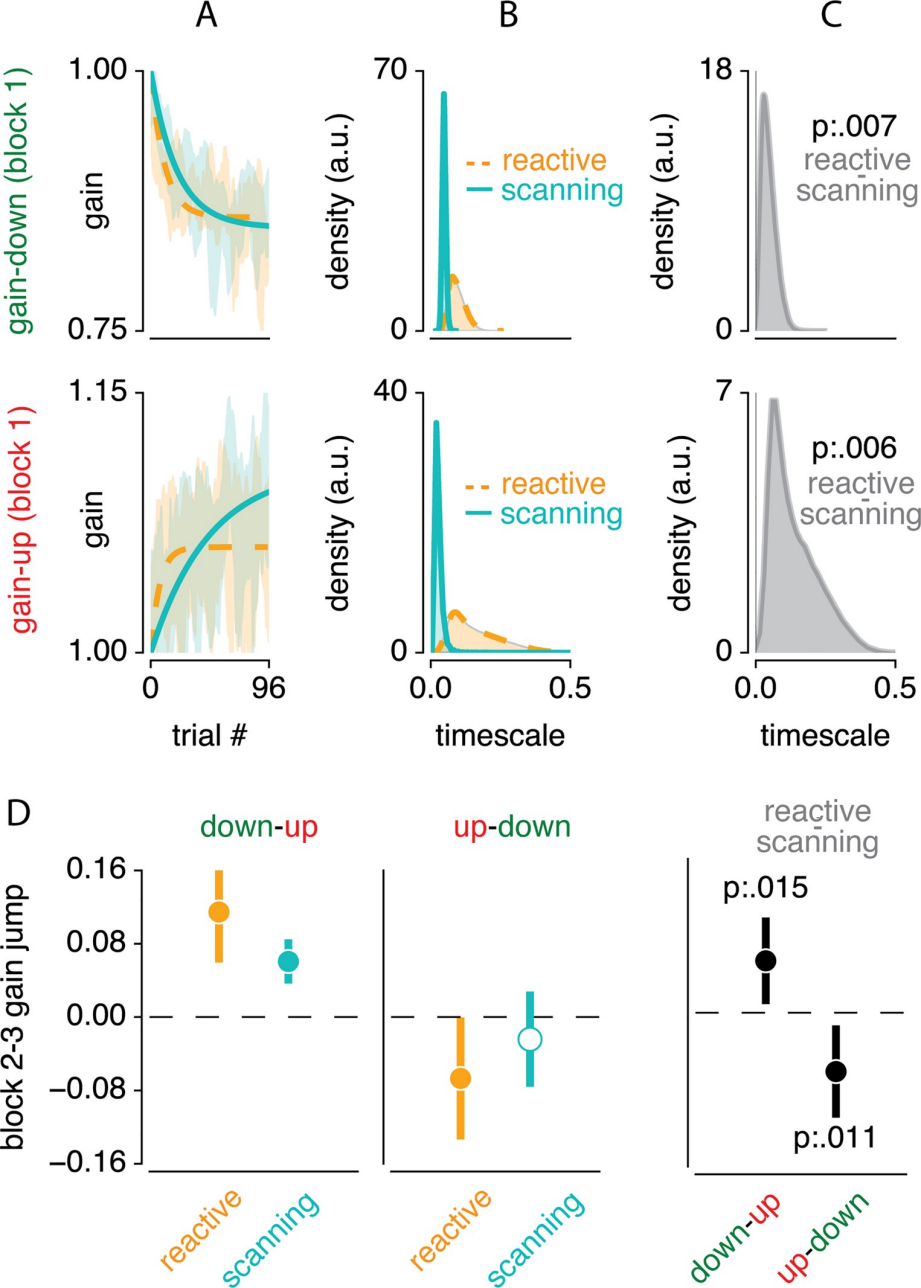
Indications for fast and slow process contributions. (A) Exponential functions fitted to the first adaptation block of each condition. (B) Bootstrapped distribution of the timescale parameter across participants, for the different conditions. (C) Difference in this timescale between reactive and scanning for both gain direction conditions. This shows that reactive compared to scanning saccade adaptation occurs at a faster rate. (D) Changes in saccade gain between the average of the last 6 saccades of the second block and the average of the first 6 saccades of the third block. Results are shown for both gain direction reversals (up-to-down and down-to-up) for both reactive and scanning saccades, and for the difference between these saccade types. This shows that when adapting reactive compared to scanning saccades, adaptation changes more quickly from one to another gain-change situation.

To quantify these observations, we first analyzed the timescale of adaptation. Fig 4 shows an exponential function fitted to the data from the second block (i.e. the first adaptation block), both for gain-up and gain-down adaptation. Indeed, the timescale of this exponential was slower for scanning compared to reactive saccades, both in the gain-down (p = .007) and gain-up (p = .006) blocks (Fig 4A and 4B). Furthermore, we tested whether adaptation changed more between the second and the third block. The magnitude of such a sudden change indicates the combination of two processes: (1) the speed of forgetting of adaptation of the previous block and (2) the speed of learning at the beginning of the new block. Confirming the visual intuition described above, we find greater changes in adaptation state between the second and third block in the reactive compared to the scanning condition, for both gain-direction reversals (quantified as the mean gain over the last and first 6 trials of each block; down-up t_(11)_ = 2.892, p = .015, Cohen's d = 0.872, up-down t_(10)_ = -3.099, p = .011, Cohen's d = -0.980, see Fig 4D; note that one subject was not included in this latter analysis as he/she did not happen to have any valid saccades in the trials analyzed; see Methods for saccade exclusion criteria). This implies that the speed of forgetting and/or the speed of learning is faster in reactive compared to scanning saccades. Together, these results show that reactive compared to scanning saccade adaptation occurs at a faster timescale.

**Fig 5:**
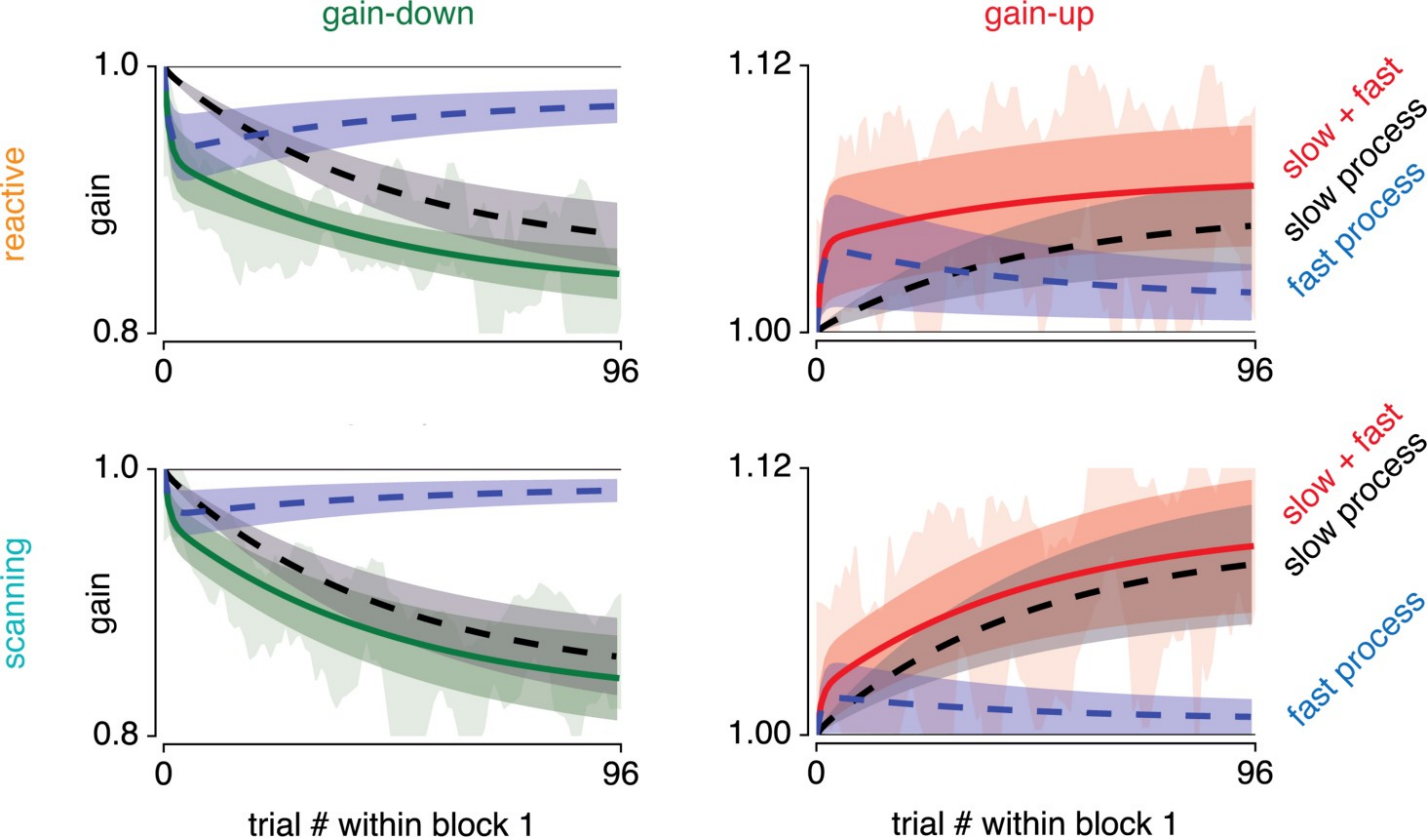
Dual-rate model fits to gain-up and gain-down adapted reactive and scanning saccades. Fitted fast (blue) and slow (black) processes to overall adaptation (green when gain-down and red when gain-up) to data from the second block. Shaded areas around model fits represent the 95% CI over participants. Underlying shaded data area indicates 95% CI over participants of moving average over six trials (as in Fig 3).

We next investigated whether the faster timescale observed above can be explained in terms of dual state decomposition into a fast and slow process [17]. For these analyses, we focused on data from the second block (i.e. first adaptation block). Fig 5 shows the fitted slow and fast processes to the data. Visual inspection of this figure suggests that the slow process contributes more to overall adaptation in scanning compared to reactive saccades.

**Fig 6:**
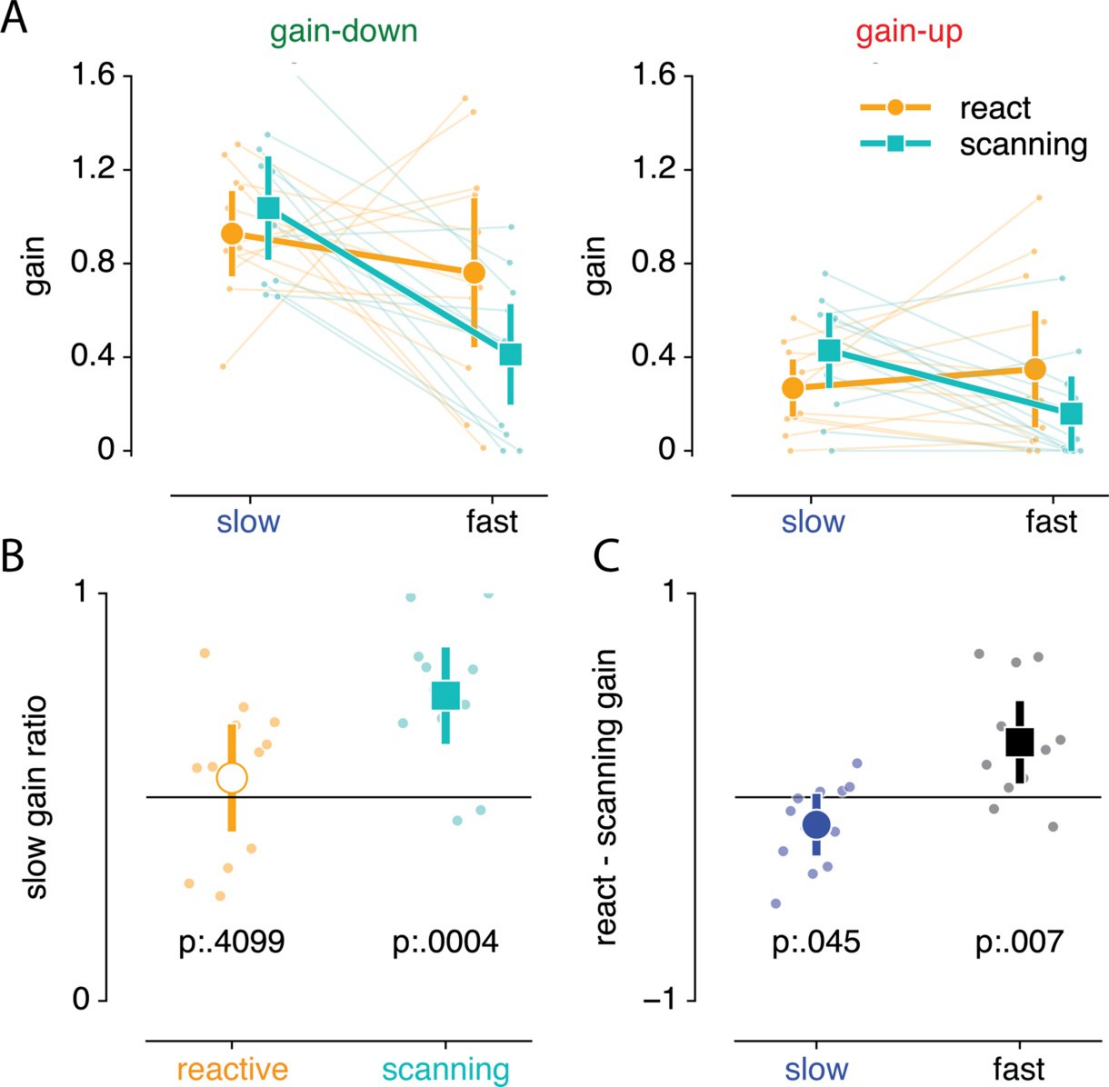
Quantification of dual process contribution to reactive and scanning saccades. (A) Comparison of process gain in both saccade type (reactive and scanning) and direction (gain-up and gain-down) blocks. (B) Contribution of slow process to overall adaptation averaged over gain-down and gain-up blocks for reactive and scanning blocks separately. This shows that reactive saccades are equally driven by the fast and slow process, whereas scanning saccades are driven mainly by the slow process. (C) Reactive minus scanning process gain averaged over gain-up and gain-down blocks for the slow and fast process separately. This shows that the slow process is stronger in scanning compared to reactive saccades, while the fast process is stronger in reactive compared to scanning saccades. Error bars depict 95% CI over participants. Individual dots and lines indicate individual participants.

The gains of both processes are summarized in Fig 6A. Overall process gain was roughly equal between reactive and scanning saccades (0.576 vs 0.509 respectively, main effect of condition F_(1,11)_ = 3.810, p = .077, *η*^2^*p* = .013). Adaptation gain was indeed much higher for gain down compared to gain up adaptation (0.784 vs 0.301 respectively , main effect of direction F_(1,11)_ = 40.342, p = 5.434 * 10^−5^, *η*^2^*p* = .650). Yet, this difference was not different between reactive and scanning saccades (interaction between condition and direction, F_(1,11)_ = 1.771, p = .210, *η*^2^*p* = .008). Also, adaptation was driven more strongly by the slow compared to the fast process (0.665 vs 0.420 respectively, main effect of process F_(1,11)_ = 12.869, p = .004, *η*^2^*p* = .167). This difference was mainly driven by gain-down as opposed to gain-up adaptation (0.396 vs 0.094 respectively, interaction beween direction and process F_(1,11)_ = 6.076, p = .031, *η*^2^*p* = .063). However, this interaction could be driven by increased overall adaptation magnitude in gain-down compared to gain-up adaptation. To account for this, we calculated the ratio of slow compared to overall (fast+slow) gain and compared this ratio between gain-up and gain-down adaptation. This showed that when controlling for overall adaptation gain, there is no differential contribution of the fast and slow processes between gain-up and gain-down adaptation (p = .667). Of particular importance to the purpose of this study, the fast and slow process contributed differently to reactive and scanning saccades (interaction between condition and process, F_(1,11)_ = 10.256, p = .008, *η*^2^*p* = .114). This differential contribution of the fast and slow process to reactive and scanning saccades was not different between gain-up/gain-down adaptation (three-way interaction between condition, direction and process, F_(1,11)_ = 0.160, p = .696, *η*^2^*p* = .002).

The main purpose of this study was to establish whether the fast or slow process contributes more to both scanning and reactive saccade adaptation. In order to investigate this, we further explored the differential contribution of the fast and slow process to overall adaptation. Since this was not different between gain-up and gain-down adaptation, we averaged over both gain direction conditions. We first analyzed the ratio of slow compared to overall (slow+fast) gain (Fig 6B). This showed that the slow and fast process contributed equally to reactive saccade adaptation (mean slow ratio of 0.547 was not different from 0.5 with t_(11)_ = 0.857, p = .410, Cohen's d = 0.258), while scanning saccade adaptation was mainly driven by the slow process (mean slow ratio of 0.749 was different from 0.5 with t_(11)_ = 5.053, p = 3.705 ∙ 10^−4^, Cohen's d = 1.523). This differential process contribution could either be due to increased slow or decreased fast process contribution in the scanning compared to reactive saccade adaptation. To investigate this, we computed the difference in adaptation gain between the reactive and scanning conditions for both fast and the slow process (Fig 6C). This showed that slow process gain is 0.135 larger in scanning compared to reactive saccade adaptation (t_(11)_ = 2.265, p = .045, Cohen's d = 0.683). Conversely, fast process gain was 0.270 larger in the reactive compared to the scanning saccade conditions (t_(11)_ = 3.267, p = .007, Cohen's d = 0.985).

In sum, these results show that scanning saccade adaptation was driven relatively more by the slow compared to the fast process, whereas reactive saccade adaptation is driven equally by fast and slow process. The difference between reactive and scanning saccade adaptation is mainly due to a decreased fast process in scanning saccades, but also by a slight increase in the slow process in reactive saccades.

**Fig 7:**
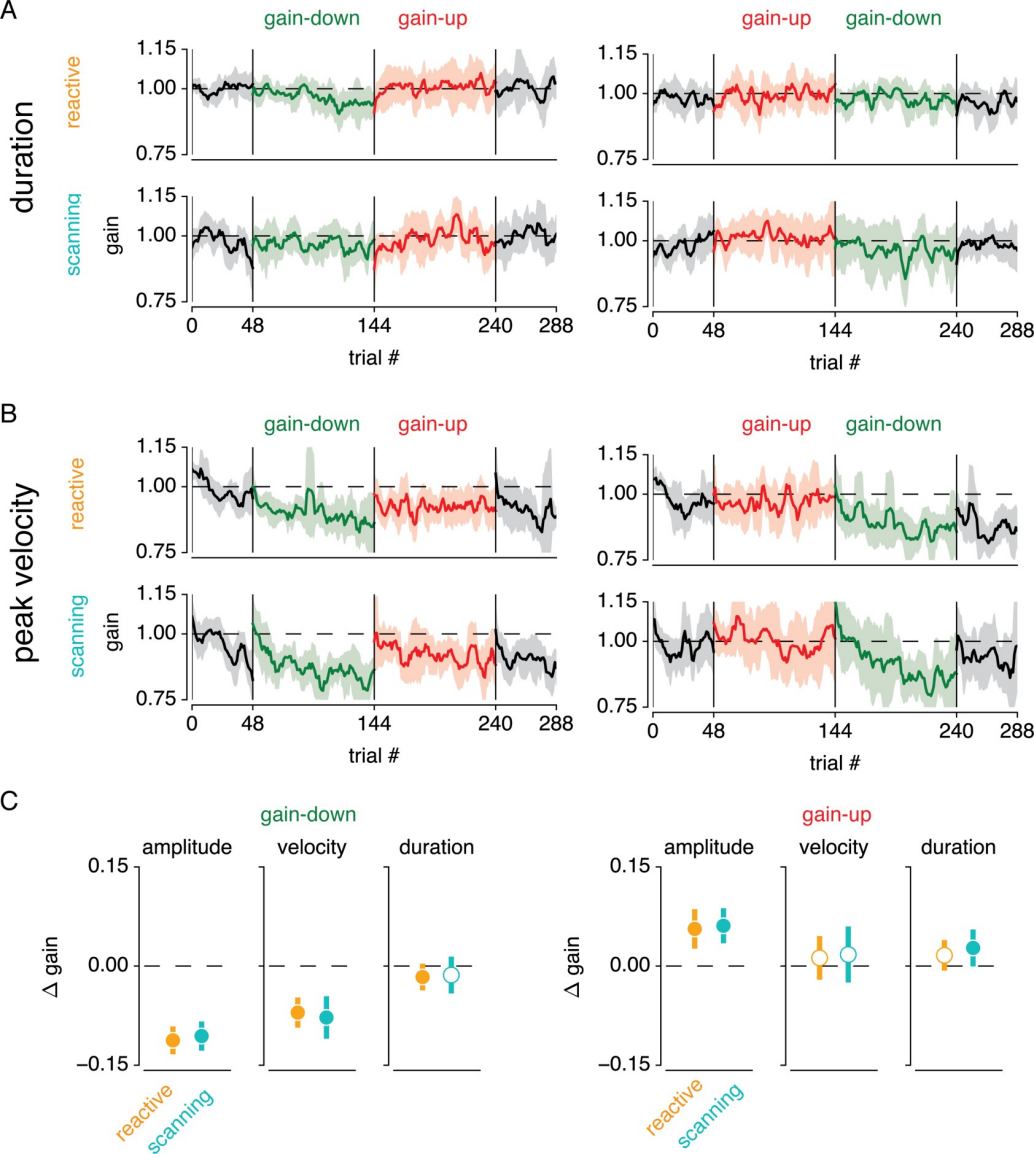
Saccade duration and peak velocity changes. Saccade duration (A) and peak velocity (B) across trials in reactive and scanning saccade adaptation, for the two different gain direction block orders (down-up and up-down). Saccade duration and peak velocity are depicted as a ratio to the median across the first block (per saccade direction, see Methods) and shown as running average over 6 saccades (the hexagonal period). Shaded areas correspond to 95% CI over participants. (C) Median saccade amplitude, peak velocity and duration over the last 16 trials of the second bock (i.e. first adaptation block). Error bars indicate 95% CI over participants.

To gain more insight into the processes underlying adaptation, we also analyzed changes in saccadic velocity and duration (see Fig 7). Fig 7A and 7B show changes in these saccade parameters over the course of the experiment. The change in these parameters is expressed in gain relative to the baseline block, in identical fashion to the results on saccade amplitude in Fig 3. Visual inspection of this figure illustrates that gain-down, but not gain-up adaptation is driven mainly by changes in peak velocity, in both reactive and scanning saccades. To quantify this, we computed the median saccade amplitude, velocity and duration change over the last 16 trials in the first adaptation block (Fig 7C). This analysis shows that absolute saccade amplitude change was stronger in gain-down compared to gain-up adaptation (0.109 vs 0.059 respectively, main effect of direction F_(1,11)_ = 21.604, p = 7.074 ∙ 10^−4^, *η*^2^*p* = .627), but that saccade amplitude change was not different between reactive and scanning saccade adaptation (0.084 vs 0.083 respectively, main effect of condition F_(1,11)_ = 0.021, p = .888, *η*^2^*p* = 1.980 ∙ 10^−4^, interaction between condition and direction, F_(1,11)_ = 0.932, p = .355, *η*^2^*p* = .008). To test the extent to which adaptation was driven by changes in peak velocity and duration, we set up two separate ANOVAs (one for gain-up and one for gain-down adaptation) with the factors of saccade parameter (2 levels: velocity or duration) and saccade type (2 levels: reactive or scanning). This showed that peak velocity change was greater than duration change during gain-down adaptation (-0.037 vs -0.008 respectively, main effect of saccade parameter F_(1,11)_ = 31.660, p = 1.541 ∙ 10^−4^, *η*^2^*p*= .385), but not during gain-up adaptation (0.007 vs 0.011 respectively, main effect of saccade parameter F_(1,11)_ = 0.195, p = .667, *η*^2^*p* = .003). This shows that gain-down adaptation is predominantly driven by changes in peak velocity, whereas gain-up adaptation is driven equally by changes in duration and velocity. This result replicates findings from the literature [33], and leads to the suggestion that gain-down adaptation is mainly driven by recalibration of the forward model, whereas gain-up adaptation is mainly driven by target remapping. Importantly, saccade velocity and duration changes were not modulated by saccade type (i.e. reactive or scanning; interaction between saccade parameter and saccade type in gain-down adaptation: F_(1,11)_ = 0.167, p = .691, *η*^2^*p* = .003, in gain-up adaptation: F_(1,11)_ = 0.053, p = .822, *η*^2^*p* = 6.203 ∙ 10^−4^). This strongly implies that error assignment was not different between reactive and scanning saccade adaptation.

**Fig 8:**
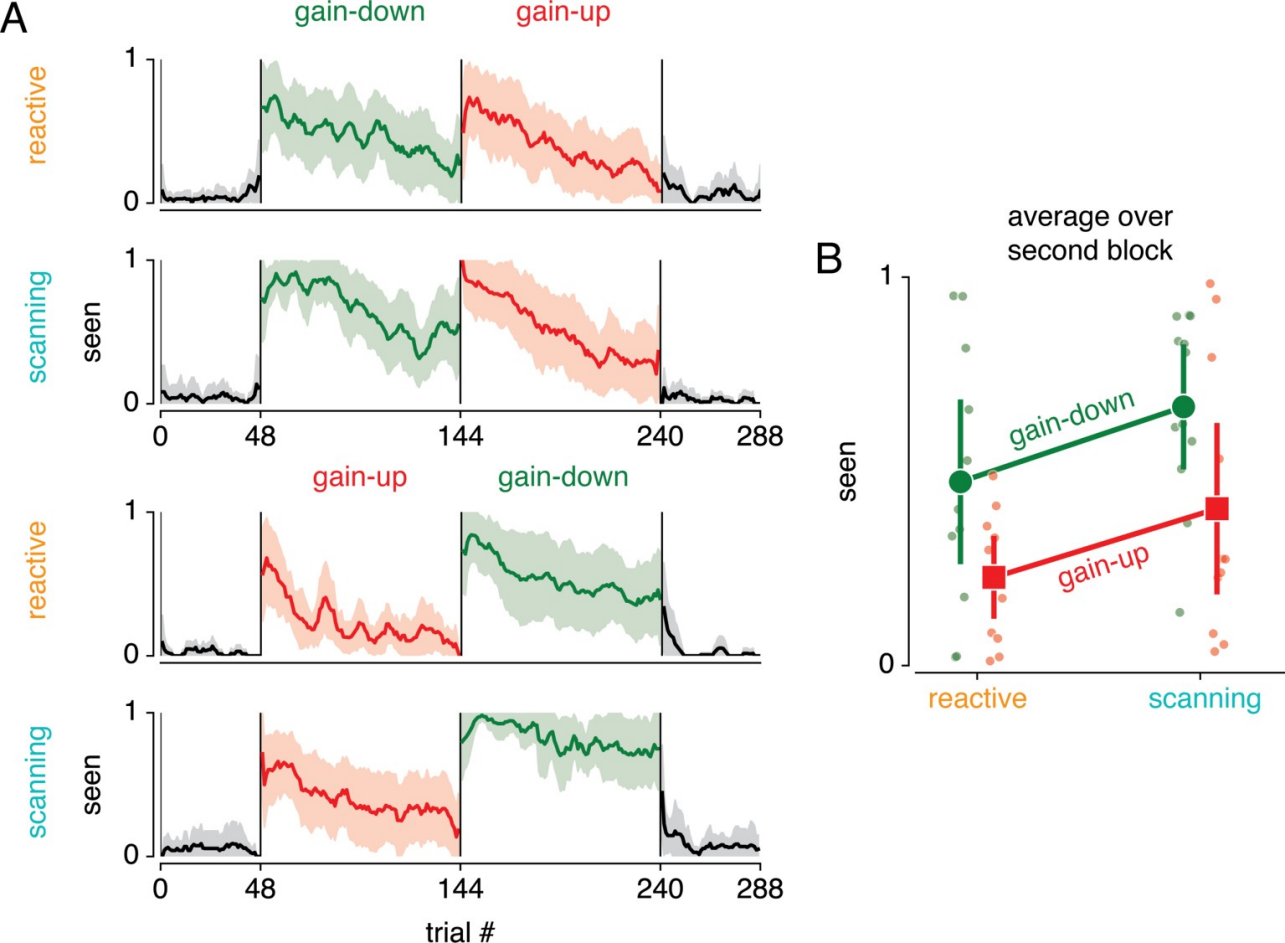
Saccade target displacement awareness. (A) Across participant average of binary displacement awareness (i.e. seen versus unseen) across the experiment, shown as running average over 6 saccades (the hexagonal period). Shaded areas correspond to 95% CI over participants. This verifies that a target displacement was not seen in the baseline blocks (i.e. blocks 1 and 4 colored black). In addition, it shows that target displacement was seen more often at the beginning compared to the end of an adaptation block. (B) Average displacements awareness per condition in the second block. The target displacement was perceived more often in gain-down compared to gain-up adaptation, and more often in scanning compared to reactive saccade conditions (see main text for statistics).

In addition to measuring eye movements, we asked subjects to report their awareness of the target displacement in a binary fashion (i.e. seen or not seen; see Methods). Fig 8A depicts this seen judgement throughout the experiment. First, this confirms that subjects generally did not report any displacements in the baseline blocks. Second, it shows that subjects saw the displacement most strongly at the beginning of each adaptation block, to then gradually become more invisible. To relate these findings to the results presented above, we further analyzed data from the second block (i.e. first adaptation block). Fig 8B summarizes the average judgements across conditions. This shows that the displacement was seen more in scanning compared to reactive saccade adaptation conditions (F_(1,11)_ = 11.954, p = .005, *η*^2^*p* = .124), and more in the gain-down compared to gain-up conditions (F_(1,11)_ = 10.204, p = .009, *η*^2^*p* = .234). There was no interaction between saccade type and gain direction (F_(1,11)_ = 0.027, p = .872, *η*^2^*p* = 2.401 ∙ 10^−4^).

## Discussion

We studied the contributions of implicit and explicit learning (indexed by the fast and slow process [17]) to reactive and scanning gain-down and gain-up saccade adaptation. First, we showed that gain-up adaptation was weaker overall compared to gain-down adaptation. Second, our results showed that scanning saccade adaptation was driven more by the slow compared to the fast process, whereas reactive saccade adaptation was driven equally strongly by the fast and slow process. This was caused mainly by an increased fast process contribution but also by a decreased slow process contribution in reactive compared to scanning saccade adaptation. Third, we found no differential fast and slow process contribution to gain-down and gain-up adaptation. Fourth, we found that gain-down adaptation was driven predominantly by peak velocity changes, while gain-up adaptation was driven equally by velocity and duration changes. This effect was not different between reactive and scanning saccade adaptation. Finally, we found that the target displacement was seen more often in scanning compared to reactive saccade adaptation and more often in gain-down compared to gain-up adaptation.

The fast and slow process were recently associated with explicit and implicit learning respectively [19,20]. Implicit learning refers to automatic learning that cannot be actively engaged or disengaged. In contrast, explicit learning refers to the voluntary and active effort to flexibly alter motor plans. This distinction shows marked similarities to the difference between reactive and scanning saccades. Specifically, implicit learning and reactive saccades are relatively automatic, whereas explicit learning and voluntary saccade generation are characterized by additional cognitive processes such as attention and working memory [9,13,14,21–26]. The main aim of the present study was to elucidate the degree to which reactive and voluntary saccades are driven by implicit and explicit learning. We suggest that learning type (i.e. explicit versus implicit) could be contingent upon the mechanisms involved in the generation of the saccade. This would have predicted that reactive saccade adaptation should mainly be driven by the slow process, while scanning saccade adaptation should mainly be driven by the fast process. Alternatively, we suggest that the increased cognitive efforts associated with voluntary saccade execution could in fact curtail remaining capacity required for explicit learning [27,28]. This would have predicted that scanning saccade adaptation would be driven less strongly by the fast process. Our results indeed agree well with this latter interpretation by showing that scanning saccade adaptation was characterized by strong slow and weak fast learning, while reactive saccade adaptation was driven equally by the slow and fast process. In addition, our results support a trade-off mechanism between explicit and implicit learning [38], as we found that both explicit learning was increased and implicit learning was decreased in reactive compared to scanning saccade adaptation. In sum, our results suggest that increasing cognitive load associated with saccade execution impairs explicit learning. The resulting reduction in overall learning is then relieved by increased implicit learning.

The differential cognitive involvement can also be viewed in terms of differential neural contributions. Specifically, both explicit learning [22,24,39–42], working memory and attention [43–45], and the generation of voluntary saccades [46–50] depend on contributions from a ‘dorsal attention network’ [45] of (pre)frontal-parietal regions (most notably the frontal eye fields and dorsolateral prefrontal cortex). Although the neural site of adaptation (rather than generation) of reactive and scanning saccades remains an active area of investigation [3,51–54], a recent study suggested that the adaptation of voluntary compared to reactive saccades places larger demands on the dorsal- compared to the ventral attention network [55]. In sum, this suggests that the demand for cognitive resources required by both voluntary saccades and explicit learning can be viewed in terms of competition for neural resources, most likely in the dorsal attention network.

This cognitive load [27,28] interpretation of our results is further supported by studies of healthy aging. Such studies generally find that senescence is associated with degrading cognitive performance [56], and disproportionate decreases in frontal lobe volume [57–59]. In addition, senescence is related to reductions in explicit but not implicit learning [60–66]. Moreover, these age-related declines in explicit learning were related to the age-related declines in cognitive performance [60,67,68]. Finally, age-related changes in voluntary saccade performance accompany age-related changes in frontal lobe maturation [69–71]. Together, this supports the notion that both voluntary saccades and explicit learning depend on availability of cognitive resources mediated by frontal cortex.

Another factor that may have influenced the relative contributions of the fast and slow process is that of error assignment. Retinal errors can either be attributed to a faulty forward model of the motor apparatus, or to motion of the target [30]. Adaptation based on the forward model should result in mid-flight changes in eye position, whereas adaptation based on target remapping results in regular saccades to a remapped position [30]. As gain-down but not gain-up adaptation was shown to be characterized by disproportionate reductions in velocity over duration [29], it was suggested that gain-down adaptation depends relatively more on adaptation of the forward model. Our data confirm this finding by showing disproportionate decreases in peak velocity over duration in gain-down but not in gain-up adaptation. Importantly however, saccade type (reactive or scanning) did not affect these differential saccade parameter changes, suggesting that error assignment was not different between reactive and scanning saccades. The issue of error assignment is particularly interesting for the present study as previous work showed that assigning credit to a faulty forward model leads to fast learning and forgetting, while assigning credit to a moved target leads to slow learning and forgetting [30]. Based on our findings on credit assignment, this predicts increased fast process contribution in gain-down compared to gain-up adaptation, and no differential fast and slow process contribution between reactive and scanning saccade adaptation. Yet, our results disconfirm both of these predictions, as we found (1) no differential fast and slow process contribution between gain-up and gain-down adaptation, and (2) greater relative slow contributions in voluntary compared to reactive saccades. This discrepancy is potentially explained by differences in saccade adaptation paradigms between previous work and ours. Specifically, the present study adapted saccade amplitude, whereas previous work adapted saccade direction [30]. Indeed, other studies investigating saccade amplitude also appear to show no clear difference in learning/forgetting speed between gain-up and gain-down adaptation [18,29]. Future work is therefore required to better understand the conditions under which error assignment leads to differential learning and forgetting speeds. Regardless, we found no differential error assignment between reactive and scanning saccades. We therefore argue that error assignment does not affect our conclusions regarding implicit and explicit learning contributions in reactive and scanning saccade adaptation.

In addition, we suggested that increased displacement awareness could lead to errors being increasingly assignment to the motion of a target [30]. However, our results run counter to this notion as we found (1) increased target awareness, but no differential error assignment between scanning compared to reactive saccades and (2) increased displacements awareness in gain-down compared to gain up adaptation (in line with [72]). This implies that errors are in fact not assigned to a ‘moved target’ [29]. Instead, it is more likely that participants did not attribute errors to a change in the external world (i.e. a moved target), but instead to an error in their internal representation of space. This suggests that adaptation here was based on distortions of internal spatial representations, rather than by remapping target positions within those representations. This interpretation agrees well with findings of altered perceptual space after gain-up but not gain-down adaptation [73].

Conversely, we suggested that increased displacement awareness could also lead to increased explicit, and therefore fast learning. However, our results showed equal contributions of fast and slow learning in gain-down compared to gain-up adaptation, although displacement awareness was greater in gain-down adaptation. In addition, even though displacement awareness was greater in scanning compared to reactive saccade adaptation, we found decreased fast learning in scanning compared to reactive saccade adaptation. These results are potentially explained by a disconnect between definition of the target displacement on the one hand, and of the saccade endpoint error on the other hand. Specifically, target displacement magnitude is given by the distance between the pre-saccadic and the post-saccadic target. Yet, saccade error magnitude is given by the distance of the post-saccadic target to the saccade landing position. In other words, when a saccade lands exactly at a displaced saccadic target position (i.e. perfect adaptation), there is no saccade error but still a considerable target displacement. One the other hand, when a saccade lands some distance away from a non-displaced saccadic target, there is considerable saccadic error but no target displacement. This disconnect between target displacement and saccade error magnitude is further exacerbated by the overrepresentation of the fovea in retinotopically organized areas. In fact, while saccadic error is always relative to fixation, target displacements are differently warped by foveal magnification depending on the direction of the jump in relation to the direction of the saccade. Specifically, the retinotopic representation of a target displacement is greater when it jumps towards versus away from the fovea. This can explain why gain-down compared to gain-up adaptation resulted in greater displacement awareness. In fact, the target jumped across the fovea in gain-down adaptation, while it moved further into the periphery in gain-up adaptation. In addition, stimuli that move in opposite direction of a saccade have a higher retinal velocity than stimuli moving in the same direction of a saccade. This means that in gain-down compared to gain-up adaptation, target displacements had a higher retinal velocity. This could have made the target displacements more salient in the gain-down conditions, in turn contributing to the increased displacement awareness in this condition. Finally, the greater displacement awareness in scanning compared to reactive saccade adaptation is likely explained by the increased visual references in the scanning saccade condition induced by all targets being presented simultaneously. In sum, this can explain why target jump awareness was disconnected to explicit learning. We suggest that future studies interested in directly measuring the explicit component of saccadic adaptation should therefore gauge explicit knowledge of the saccadic endpoint error rather than awareness of the target jump.

Finally, we employed a global saccadic adaptation paradigm, as this was shown to be a more potent adaptation paradigm compared to one- or two-way adaptation protocols [31]. In one- and two-way adaptation, learning is specific to the adapted saccade vectors, and does not generalize to other saccade directions. In contrast, global adaptation generalizes equally to all non-adapted saccade directions, pointing to a general saccade gain changing mechanism. This global adaptation resulted in overall faster adaptation compared to vector-specific adaptation [31]. It is therefore possible that the overall magnitude of the fast process depends on the saccade adaptation paradigm. However, the main aim of this study was to ascertain relative differences in fast and slow process contributions between reactive and scanning saccades. We therefore argue that our conclusions regarding implicit and explicit learning are not specific to the global adaptation paradigm.

In sum, our results suggest that increasing cognitive load associated with movement execution reduces the capacity for explicit learning. In general, this suggests that increasingly simplified training environments will enhance performance particularly when little time for learning is available. Conversely, in situations that place great demands on cognitive resources, learning will be mainly based on implicit learning and thus will take time to develop.

## References

[1] McLaughlin SC. Parametric adjustment in saccadic eye movements. Percept Psychophys. 1967;2: 359–362. 10.3758/BF03210071

[2] Deubel H. Separate adaptive mechanisms for the control of reactive and volitional saccadic eye movements. Vision Res. Elsevier; 1995;35: 3529–3540. 8560817 10.1016/0042-6989(95)00058-m

[3] Pelisson D, Alahyane N, Panouillères M, Tilikete C. Sensorimotor adaptation of saccadic eye movements. Neurosci Biobehav Rev. 2010;34: 1103–1120. 10.1016/j.neubiorev.2009.12.010 20026351

[4] Deubel H, Koch C, Bridgeman B. Landmarks facilitate visual space constancy across saccades and during fixation. Vision Res. 2010;50: 249–259. 10.1016/j.visres.2009.09.020 19833147

[5] Alahyane N, Salemme R, Urquizar C, Cotti J, Guillaume A, Vercher J-L, et al Oculomotor plasticity: Are mechanisms of adaptation for reactive and voluntary saccades separate? Brain Res. 2007;1135: 107–121. 10.1016/j.brainres.2006.11.077 17210146

[6] Cotti J, Panouillères M, Munoz DP, Vercher J-L, Pélisson D, Guillaume A. Adaptation of reactive and voluntary saccades: different patterns of adaptation revealed in the antisaccade task. J Physiol-London. Blackwell Publishing Ltd; 2009;587: 127–138. 10.1113/jphysiol.2008.159459 19015199 PMC2670028

[7] Fujita M, Amagai A, Minakawa F, Aoki M. Selective and delay adaptation of human saccades. Brain Res Cogn Brain Res. 2002;13: 41–52. 10.1016/S0926-6410(01)00088-X 11867249

[8] Cotti J, Guillaume A, Alahyane N, Pélisson D, Vercher J-L. Adaptation of voluntary saccades, but not of reactive saccades, transfers to hand pointing movements. J Neurophysiol. 2007;98: 602–612. 10.1152/jn.00293.2007 17553949

[9] Hutton SB. Cognitive control of saccadic eye movements. Brain Cognition. 2008;68: 327–340. 10.1016/j.bandc.2008.08.021 19028265

[10] Rizzolatti G, Riggio L, Dascola I, Umiltá C. Reorienting attention across the horizontal and vertical meridians: evidence in favor of a premotor theory of attention. Neuropsychologia. 1987;25: 31–40. 10.1016/0028-3932(87)90041-8 3574648

[11] Schneider WX. VAM: A neuro-cognitive model for visual attention control of segmentation, object recognition, and space-based motor action. Visual Cognition. 1995;2: 331–376. 10.1080/13506289508401737

[12] Wollenberg L, Deubel H, Szinte M. Visual attention is not deployed at the endpoint of averaging saccades. Pack C, editor. PLoS Biol. Public Library of Science; 2018;16: e2006548 10.1371/journal.pbio.2006548 29939986 PMC6034887

[13] Roberts RJ, Hager LD, Heron C. Prefrontal cognitive processes: Working memory and inhibition in the antisaccade task. J Exp Psychol Gen. 1994;123: 374–393. 10.1037//0096-3445.123.4.374

[14] Kane MJ, Bleckley MK, Conway AR, Engle RW. A controlled-attention view of working-memory capacity. J Exp Psychol Gen. 2001;130: 169–183. 11409097 10.1037//0096-3445.130.2.169

[15] Kojima Y, Iwamoto Y, Yoshida K. Memory of learning facilitates saccadic adaptation in the monkey. J Neurosci. Society for Neuroscience; 2004;24: 7531–7539. 10.1523/JNEUROSCI.1741-04.2004 15329400 PMC6729647

[16] Miall RC, Jenkinson N, Kulkarni K. Adaptation to rotated visual feedback: a re-examination of motor interference. Exp Brain Res. 2004;154: 201–210. 10.1007/s00221-003-1630-2 14608451

[17] Smith MA, Ghazizadeh A, Shadmehr R. PLOS Biology: Interacting Adaptive Processes with Different Timescales Underlie Short-Term Motor Learning. PLoS Biol. 2006;4: e179 10.1371/journal.pbio.0040179 16700627 PMC1463025

[18] Ethier V, Zee DS, Shadmehr R. Spontaneous Recovery of Motor Memory During Saccade Adaptation. J Neurophysiol. 2008;99: 2577–2583. 10.1152/jn.00015.2008 18353917 PMC2733835

[19] McDougle SD, Bond KM, Taylor JA. Explicit and Implicit Processes Constitute the Fast and Slow Processes of Sensorimotor Learning. J Neurosci. Society for Neuroscience; 2015;35: 9568–9579. 10.1523/JNEUROSCI.5061-14.2015 26134640 PMC4571499

[20] Huberdeau DM, Krakauer JW, Haith AM. Dual-process decomposition in human sensorimotor adaptation. Curr Opin Neurobiol. 2015;33: 71–77. 10.1016/j.conb.2015.03.003 25827272

[21] Taylor JA, Ivry RB. Flexible cognitive strategies during motor learning. Diedrichsen J, editor. PLoS Comput Biol. 2011;7: e1001096 10.1371/journal.pcbi.1001096 21390266 PMC3048379

[22] McDougle SD, Ivry RB, Taylor JA. Taking Aim at the Cognitive Side of Learning in Sensorimotor Adaptation Tasks. Trends Cogn Sci. Elsevier Current Trends; 2016;20: 535–544. 10.1016/j.tics.2016.05.002 27261056 PMC4912867

[23] Bo J, Seidler RD. Visuospatial working memory capacity predicts the organization of acquired explicit motor sequences. J Neurophysiol. 2009;101: 3116–3125. 10.1152/jn.00006.2009 19357338 PMC2694099

[24] Anguera JA, Reuter-Lorenz PA, Willingham DT, Seidler RD. Contributions of spatial working memory to visuomotor learning. J Cognitive Neurosci. 3rd ed. 2010;22: 1917–1930. 10.1162/jocn.2009.21351 19803691

[25] Taylor JA, Thoroughman KA. Motor adaptation scaled by the difficulty of a secondary cognitive task. PLoS ONE. 2008;3: e2485 10.1371/journal.pone.0002485 18560546 PMC2413425

[26] Eversheim U, Bock O. Evidence for processing stages in skill acquisition: a dual-task study. Learn Mem. 2001;8: 183–189. 10.1101/lm.39301 11533221 PMC311376

[27] Sweller J, van Merrienboer JJG, Paas FGWC. Cognitive Architecture and Instructional Design. Educational Psychology Review. 1998;10: 251–296. 10.1023/A:1022193728205

[28] Sweller J. Cognitive Load During Problem Solving: Effects on Learning. Cognitive Science. 1988;12: 257–285. 10.1207/s15516709cog1202_4

[29] Ethier V, Zee DS, Shadmehr R. Changes in control of saccades during gain adaptation. J Neurosci. Society for Neuroscience; 2008;28: 13929–13937. 10.1523/JNEUROSCI.3470-08.2008 19091981 PMC2632981

[30] Chen-Harris H, Joiner WM, Ethier V, Zee DS, Shadmehr R. Adaptive control of saccades via internal feedback. J Neurosci. Society for Neuroscience; 2008;28: 2804–2813. 10.1523/JNEUROSCI.5300-07.2008 18337410 PMC2733833

[31] Rolfs M, Knapen T, Cavanagh P. Global saccadic adaptation. Vision Res. 2010;50: 1882–1890. 10.1016/j.visres.2010.06.010 20600235

[32] Alahyane N, Pélisson D. Retention of saccadic adaptation in humans. Ann NY Acad Sci. 2005;1039: 558–562. 10.1196/annals.1325.067 15827022

[33] Engbert R, Mergenthaler K. Microsaccades are triggered by low retinal image slip. Proc Natl Acad Sci USA. National Academy of Sciences; 2006;103: 7192–7197. 10.1073/pnas.0509557103 16632611 PMC1459039

[34] Straube A, Fuchs AF, Usher S, Robinson FR. Characteristics of saccadic gain adaptation in rhesus macaques. J Neurophysiol. 1997;77: 874–895. 10.1152/jn.1997.77.2.874 9065856

[35] Miller JM, Anstis T, Templeton WB. Saccadic plasticity: parametric adaptive control by retinal feedback. J Exp Psychol Human. 1981;7: 356–366. 10.1037//0096-1523.7.2.356 6453929

[36] Noto CT, Watanabe S, Fuchs AF. Characteristics of simian adaptation fields produced by behavioral changes in saccade size and direction. J Neurophysiol. Am Physiological Soc; 1999;81: 2798–2813. 10.1152/jn.1999.81.6.2798 10368398

[37] Robinson FR, Noto CT, Bevans SE. Effect of visual error size on saccade adaptation in monkey. J Neurophysiol. 2003;90: 1235–1244. 10.1152/jn.00656.2002 12711711

[38] Benson BL, Anguera JA, Seidler RD. A spatial explicit strategy reduces error but interferes with sensorimotor adaptation. J Neurophysiol. 2011;105: 2843–2851. 10.1152/jn.00002.2011 21451054 PMC3118744

[39] Shadmehr R, Holcomb HH. Neural correlates of motor memory consolidation. Science. 1997;277: 821–825. 9242612 10.1126/science.277.5327.821

[40] Anguera JA, Russell CA, Noll DC, Seidler RD. Neural correlates associated with intermanual transfer of sensorimotor adaptation. Brain Res. 2007;1185: 136–151. 10.1016/j.brainres.2007.09.088 17996854

[41] Seidler RD, Bo J, Anguera JA. Neurocognitive contributions to motor skill learning: the role of working memory. J Mot Behav. 2012;44: 445–453. 10.1080/00222895.2012.672348 23237467 PMC3534841

[42] Taylor JA, Ivry RB. Cerebellar and Prefrontal Cortex Contributions to Adaptation, Strategies, and Reinforcement Learning. Prog Brain Res. Elsevier; 2014;210: 217–253. 10.1016/B978-0-444-63356-9.00009-1 24916295 PMC4118688

[43] Duncan J. The multiple-demand (MD) system of the primate brain: mental programs for intelligent behaviour. Trends Cogn Sci. 2010;14: 172–179. 10.1016/j.tics.2010.01.004 20171926

[44] Corbetta M, Akbudak E, Conturo TE, Snyder AZ, Ollinger JM, Drury HA, et al A common network of functional areas for attention and eye movements. Neuron. 1998;21: 761–773. 10.1016/S0896-6273(00)80593-0 9808463

[45] Corbetta M, Shulman GL. Control of goal-directed and stimulus-driven attention in the brain. Nat Rev Neurosci. Nature Publishing Group; 2002;3: 201–215. 10.1038/nrn755 11994752

[46] Pierrot-Deseilligny C, Milea D, Müri RM. Eye movement control by the cerebral cortex. Current Opinion in Neurology. 2004;17: 17 15090873 10.1097/00019052-200402000-00005

[47] Müri RM, Nyffeler T. Neurophysiology and neuroanatomy of reflexive and volitional saccades as revealed by lesion studies with neurological patients and transcranial magnetic stimulation (TMS). Brain Cognition. 2008;68: 284–292. 10.1016/j.bandc.2008.08.018 18845373

[48] McDowell JE, Dyckman KA, Austin BP, Clementz BA. Neurophysiology and neuroanatomy of reflexive and volitional saccades: Evidence from studies of humans. Brain Cognition. Academic Press; 2008;68: 255–270. 10.1016/j.bandc.2008.08.016 18835656 PMC2614688

[49] Munoz DP, Everling S. Look away: the anti-saccade task and the voluntary control of eye movement. Nat Rev Neurosci. Nature Publishing Group; 2004;5: 218–228. 10.1038/nrn1345 14976521

[50] Schall JD. Visuomotor Functions in the Frontal Lobe. Annu Rev Vis Sci. 2015;1: 469–498. 10.1146/annurev-vision-082114-035317 28532381

[51] Hopp JJ, Fuchs AF. The characteristics and neuronal substrate of saccadic eye movement plasticity. Prog Neurobiol. 2004;72: 27–53. 10.1016/j.pneurobio.2003.12.002 15019175

[52] Panouillères M, Habchi O, Gerardin P, Salemme R, Urquizar C, Farne A, et al A Role for the Parietal Cortex in Sensorimotor Adaptation of Saccades. Cereb Cortex. 2014;24: 304–314. 10.1093/cercor/bhs312 23042755

[53] Bender J, Tark K-J, Reuter B, Kathmann N, Curtis CE. Differential roles of the frontal and parietal cortices in the control of saccades. Brain Cognition. 2013;83: 1–9. 10.1016/j.bandc.2013.06.005 23867736 PMC3954743

[54] Zimmermann E, Lappe M. Visual Space Constructed by Saccade Motor Maps. Front Hum Neurosci. 2016;10: 595 10.3389/fnhum.2016.00595 27242488 PMC4870275

[55] Gerardin P, Miquée A, Urquizar C, Pélisson D. Functional activation of the cerebral cortex related to sensorimotor adaptation of reactive and voluntary saccades. NeuroImage. Elsevier; 2012;61: 1100–1112. 10.1016/j.neuroimage.2012.03.037 22465298

[56] Craik FI, Salthouse TA. Handbook of Aging and Cognition II Mahwah: Lawrence Erlbaum; 2000.

[57] Raz N, Lindenberger U, Rodrigue KM, Kennedy KM, Head D, Williamson A, et al Regional brain changes in aging healthy adults: general trends, individual differences and modifiers. Cereb Cortex. 2005;15: 1676–1689. 10.1093/cercor/bhi044 15703252

[58] Raz N, Gunning FM, Head D, Dupuis JH, McQuain J, Briggs SD, et al Selective aging of the human cerebral cortex observed in vivo: differential vulnerability of the prefrontal gray matter. Cereb Cortex. 1997;7: 268–282. 9143446 10.1093/cercor/7.3.268

[59] Allen JS, Bruss J, Brown CK, Damasio H. Normal neuroanatomical variation due to age: The major lobes and a parcellation of the temporal region. Neurobiology of Aging. Elsevier; 2005;26: 1245–1260. 10.1016/j.neurobiolaging.2005.05.023 16046030

[60] Bock O. Components of sensorimotor adaptation in young and elderly subjects. Exp Brain Res. 2005;160: 259–263. 10.1007/s00221-004-2133-5 15565436

[61] Bock O, Girgenrath M. Relationship between sensorimotor adaptation and cognitive functions in younger and older subjects. Exp Brain Res. 2006;169: 400–406. 10.1007/s00221-005-0153-4 16328306

[62] Fernández-Ruiz J, Hall C, Vergara P, Díiaz R. Prism adaptation in normal aging: slower adaptation rate and larger aftereffect. Brain Res Cogn Brain Res. 2000;9: 223–226. 10.1016/S0926-6410(99)00057-9 10808133

[63] Buch ER, Young S, Contreras-Vidal JL. Visuomotor adaptation in normal aging. Learn Mem. 2003;10: 55–63. 10.1101/lm.50303 12551964 PMC196655

[64] McNay EC, Willingham DB. Deficit in learning of a motor skill requiring strategy, but not of perceptuomotor recalibration, with aging. Learn Mem. 1998;4: 411–420. 10701880 10.1101/lm.4.5.411

[65] Heuer H, Hegele M, Sülzenbrück S. Implicit and explicit adjustments to extrinsic visuo-motor transformations and their age-related changes. Hum Mov Sci. 2011;30: 916–930. 10.1016/j.humov.2010.07.004 20934231

[66] Heuer H, Hegele M. Generalization of implicit and explicit adjustments to visuomotor rotations across the workspace in younger and older adults. J Neurophysiol. 2011;106: 2078–2085. 10.1152/jn.00043.2011 21775724

[67] Anguera JA, Reuter-Lorenz PA, Willingham DT, Seidler RD. Failure to engage spatial working memory contributes to age-related declines in visuomotor learning. J Cognitive Neurosci. 2011;23: 11–25. 10.1162/jocn.2010.21451 20146609

[68] Langan J, Seidler RD. Age differences in spatial working memory contributions to visuomotor adaptation and transfer. Behav Brain Res. 2011;225: 160–168. 10.1016/j.bbr.2011.07.014 21784106 PMC3170505

[69] Munoz DP, Broughton JR, Goldring JE, Armstrong IT. Age-related performance of human subjects on saccadic eye movement tasks. Exp Brain Res. 1998;121: 391–400. 9746145 10.1007/s002210050473

[70] Fischer B, Biscaldi M, Gezeck S. On the development of voluntary and reflexive components in human saccade generation. Brain Res. 1997;754: 285–297. 9134986 10.1016/s0006-8993(97)00094-2

[71] Fukushima J, Hatta T, Fukushima K. Development of voluntary control of saccadic eye movements: I. Age-related changes in normal children. Brain and Development. Elsevier; 2000;22: 173–180. 10.1016/S0387-7604(00)00101-7 10814900

[72] Joosten ERM, Collins T. Probing transsaccadic correspondence with reverse correlation. J Vis. 2018;18: 10 10.1167/18.3.10 29677323

[73] Zimmermann E, Lappe M. Motor signals in visual localization. J Vis. Association for Research in Vision and Ophthalmology; 2010;10. 10.1167/10.6.2 20884551

